# A Case of Aortic Aneurism, and One of Aortic Endarteritis, with Autopsies

**Published:** 1881-05

**Authors:** B. C. Gudden

**Affiliations:** House Physician, Cook County Hospital


### Clinical Reports.

### NOTES FROM HOSPITAL PRACTICE.

### ARTICLE IV.

### A CASE OF AORTIC ANEURISM, AND ONE OF AORTIC ENDARTERITIS, WITH AUTOPSIES. By B. C. GUDDEN, M.D., House Physician, Cook County Hospital.

I. *Aortic Aneurism.* C. S., æt. thirty-eight, German; admitted to hospital December 25, 1880. Had acute rheumatism in 1875. Six weeks previous to admission he was taken with a sudden, sharp, lancinating pain in left axillary space, with slight cough. Pain increased in deep inspiration. From that time his appetite was poor, tongue furred, bowels constipated. Pain constant in the left side. Physical examination showed a loss of expansion and of respiratory movement with absence of vocal fremitus over the left lung, except in the infra-clavicular region. Flat percussion note. The right side of the thorax afforded low-pitched sounds with increased respiratory movements. Area of cardiac dullness increased, apex beat slightly moved to the right. Systolic murmur at the base, with a considerable bruit transmitted to the aorta. Liver displaced downward. Feet slightly œdematous. Treatment with ergot, iodide of potassium, counter-irritation, and anodynes.

January 7, 1881, one ounce of clear serous fluid was aspired from the left pleural cavity in the seventh intercostal space. January 14 no pulsation was felt in left radial, nor in left carotid artery. Patient was failing fast, had orthopnœa, annoying cough, and expectorated muco-purulent matter streaked with blood. Pain increased. January 21, patient had a slight chill.

Pulse 114, temperature 102.5. Quinine was administered. January 24, more bloody serum was aspired. Temperature subsided but raised to 103 January 30, when patient expectorated from four to six ounces of bright blood. Had another profuse hæmoptysis February 1, 4 a. m. Treatment, ergot and whisky, and inhalation of ammonia. February 2, pulsation ceased in the right radial artery. Patient died in the night.

Post-mortem forty-eight hours after death. Intercostal spaces of the left side bulging. A puncture gave a free exit to a large quantity of sero-sanguineous fluid. On removing the sternum, the left pleural cavity is found distended with sanious fluid. Left lung pressed upward. Right lung normal or slightly emphysematous. Pericardial fluid contained some fresh lymph and was slightly increased. Heart normal. There was about a gallon of fluid in left pleura. Large clots of blood and shreds of lymph settled at the bottom of the fluid. The pleura was ruptured near the third dorsal vertebra, near its reflection over the left lung. Removing the left lung, trachea, heart and aorta *en masse*, we found that the bodies of the second, third and fourth dorsal vertebræ had been absorbed. Mucous membrane of trachea ulcerated, and the cartilaginous rings absorbed. There was a commucation between the left bronchi and the pleural cavity through the rupture above mentioned. Then the aorta was laid open from behind into what proved to be a large *aneurismal dilatation*, which had opened into the pleural cavity. It was partly filled with fibrinous laminæ at its periphery, and these occluded the mouths of the left carotid and subclavian artery. The left lung was completely consolidated. The pressure of the aneurism had caused the absorption of the bronchial walls, as well as that of the bodies of the vertebræ.

II. *Valvular Heart Disease and Aortic Endarteritis.* O. D., æt. forty-two, American, painter, admitted to the hospital February 15, 1881. Patient had typhoid fever ten years ago, and has had several attacks of rheumatism. Had angina pectoris four years ago. January last he had for the first time a dry and hacking cough, with expectoration of a whitish, frothy mucus, which soon became purulent. It has been occasionally streaked with blood lately. On admission he had anorexia, constipation, a frequent hammering pulse, and complained of some lumbar pain. Tongue furred; cardiac area increased; apex beat in the mammary line. A blowing sound is heard at the base of the heart with both the systole and the diastole, and the systolic murmur is heard at the apex. In the right infra-scapular and infra-axillary regions, the vocal fremitus is diminished, and friction sounds are heard. Percussion note is flat. There was some œdema of feet, and the urine contained seven per cent. of albumen. Has hæmorrhoids. Treatment consisted in a nutritious diet, morphine and chloride of ammonium; to allay the cough, small doses of iodide of potassium. February 18, 1881, patient had dizziness and headache. The veins of the neck were engorged, and scarcely any pulsation could be detected in the left carotid and the left radial artery; dyspnœa and difficulty in swallowing; sharp and lancinating pain in the chest, extending to the left shoulder; slight friction sounds at top of left lung. March 3, free expectoration of blood; œdema of feet increases; patient is failing. He expectorated more blood the 15th, and died the 18th, apparently from exhaustion and anæmia. Morphine was given to relieve the pain, as often as necessary; temperature had been normal; auscultation over the base of the heart had detected no decided aneurismal bruit.

Necropsy forty-eight hours after death. On opening the thoracic cavity, both pleuræ contained one and a half pints of clear fluid. There were two ounces of clear serum in the pericardium. Heart increased in size; aortic valves thickened and shortened, the left being also perforated by an ulcerative endocarditis; fatty degeneration of the muscular substance of the heart; both lungs are œdematous; the bronchi contain some mucous matter, and their mucous membrane is injected and ecchymotic; endarteritis deformans in the arch of the aorta without dilatation; considerable narrowing of the mouths of left carotid and subclavian arteries, which are obstructed by calcareous deposits; spleen enlarged and sclerotic; liver is normal in size, but has a nutmeg appearance; sclerosis of the kidneys is well marked.

